# The median time to stopover exclusive breastfeeding among employed and unemployed mothers of infants aged 6–12 months in Ethiopia, 2019

**DOI:** 10.1038/s41598-023-29729-z

**Published:** 2023-04-17

**Authors:** Emebet Adugnaw, Getu Gizaw, Merom Girma, Getachew Arage, Mebratu Libanos, Wondosen Addis Emrie, Sewnet Sisay Chanie, Ermias Sisay Chanie

**Affiliations:** 1 Mizan Tepi University, Mizan, Ethiopia; 2 Jimma University, Jimma, Ethiopia; 3 DebreTabor University, DebreTabor, Ethiopia; 4 Debre Birhan University, Debre Birhan, Ethiopia

**Keywords:** Health care, Medical research, Risk factors

## Abstract

Early discontinuation of breastfeeding is known to be associated with avoidable childhood morbidity and mortality. The effect of maternal employment on the duration of exclusive breastfeeding and its determinants has not been addressed adequately in in Ethiopia in general and in the stud area in particular. Hence, this study was aimed to compare the time to stop exclusive breastfeeding and its determinants among employed and unemployed mothers of infants 6–12 months of age. A community-based comparative cross-sectional study was conducted from March 1 to 30, 2019. A total of 426 mothers were recruited using a simple random sampling technique. The Kaplan–Meier curve with log-rank test was used to compare the difference in cessation of exclusive breastfeeding before 6 months. Bivariate and Cox proportional hazards model were computed. Hazard ratios and their 95% confidence intervals were computed to determine the level of significance. Four hundred twenty-six (213 employed and 213 un-employed) mothers were included in the final analysis. The median duration of exclusive breastfeeding was 4 months and 6 months for infants of employed and unemployed mothers, respectively. The likelihood of ceasing of exclusive breastfeeding before 6 months of age was significantly associated with family support of exclusive breastfeeding [AHR = 3.99, 95% CI (1.9, 8.3)], and lack of exclusive breastfeeding counseling during postnatal care [AHR = 7.76, 95% CI (2.99, 20.1)], primipara mothers [AHR = 1.5, 95% CI (1.14, 2.04)], maternity leave of 4 months [AHR = 7, 95% CI 2.2, 22.2)] and employed mothers [AHR = 3.77, 95% CI (2.4, 5.9)]. The median duration of exclusive breastfeeding was shorter among employed mothers than un-employed mothers. It is clear from this study that cessation of exclusive breastfeeding was associated with the duration of paid maternity leave for employed mothers. Family support and perceived adequacy of breast milk were associated with cessation of exclusive breastfeeding before 6 months among unemployed mothers.

## Introduction

World Health Organization (WHO) defines exclusive breastfeeding as feeding an infant with only breast milk for the first 6 months of life, excluding solids or any other fluids including infant formulas except medicines, vitamins and minerals^1^.

The shorter duration of exclusive breastfeeding is not widespread in both low and high-income countries^2^. Only 37% of infants younger than 6 months are exclusively breastfed in low-income and middle-income countries^3^. In Ethiopia, the prevalence of exclusive breastfeeding 60.9%^4^.

The consequences of early discontinuation of breastfeeding include poor school performance, reduced productivity in the long run and cognitive impairment^5,6^. Moreover, it also contributes to childhood mortality and morbidity. Worldwide, suboptimal breastfeeding practices attributes to 11.6% of mortality among children younger than five years of age. Infants younger than 6 months who are not breastfed are 3–4 times more likely to die than those who received breast milk^7^.

Evidence shows that maternal employment during the first 6 months is an important factor for shorter duration discontinuation and low rates of exclusive breastfeeding^8–10^. This is because the child-caring time is mainly affected by maternal; employment status^11^. As a result of increased household income demand, maternal employment has increased rapidly^12^. Consequently, most mothers spend significant amounts of time separated from their children during the first year of life^6^. Observational studies revealed a difference of 21–27% between employed and unemployed mothers on the practice of exclusive breastfeeding^13,14^. The longer the length of the mother's working hours, the less likely the mothers breastfed their children for at least 4 months^15^. In contrast, mothers who self-defined as a housewife or as unemployed were more likely to practice exclusive breastfeeding than employed mothers^16^.

Countries that provide mothers with paid maternity leave greater than 6 months, with the availability of breastfeeding breaks, accessibility of quality infant care near the workplace and availability of facilities for pumping or storing milk have the highest exclusive breastfeeding duration and rates^10,14^.

Given the above, the impact of maternal employment on the duration of exclusive breastfeeding and its determinants has not been addressed well in Ethiopia in general and in the stud area in particular. Hence, this study was aimed to compare the duration of exclusive breastfeeding among employed and non-employed mothers and its determinants.

## Methods and materials

### Study setting, design and period

The study was conducted in Debre tabor town, which is found in the South Gondar zone, Amhara Regional State, Ethiopia. Based on the 2007 national census conducted by the Central Statistical Agency of Ethiopia (CSA), the town has a total population of 55,596, of which, 27,644 are men and 27,952 women. The town is divided into six kebeles. A community-based comparative cross-sectional study design was conducted from March 1 to 30/2019.

### Source and study population

All mothers of infants aged 6–12 months in Debre Tabor town was considered as a source population and the study population was all randomly selected employed and unemployed mothers of 6–12 months of age infants.

The sample size was calculated by Epi info statistical software version 7 using two population proportion formula. It was by calculated taking the prevalence of exclusive breastfeeding among employed and unemployed mothers 44%, 65%, respectively from a similar study, which gives the largest sample size^13^. With assumptions of 95% confidence level, a power of 80%, a designing effect of 2, and a 10% non-response rate, the ratio of employed to unemployed 1:1. Hence, the final sample size was 426 (213 employed and 213 unemployed mothers). A simple random sampling technique was used to recruit participants.

### Measurements

The outcome variable was time to stop exclusive breastfeeding. the independent variables include socio-demographic and economic characteristics, breastfeeding practices, maternal perception on EBF, health service-related factors, obstetrics and gynecologic factors, workplace breastfeeding support and dietary diversity of mothers. Exclusive breastfeeding was feeding an infant with only breast milk for the first 6 months of life, excluding solids or any other fluids including infant formulas) except for medicines, vitamins and minerals. Exclusive breastfeeding duration was defined as the time interval from the start of feeding breast milk to the introduction of any non-breast milk foods. Mothers were asked "what was the age of your child in a month when you first gave food items other than your breast milk including water. Employment: any type of employment outside the home/that takes mothers outside the home, and earn wages or salaries were defined as employment and unemployment means being a housewife/ involved in other work inside the home. Dietary diversity score (DDS) was categorized into low DDS (consumption of less than 4 food groups), medium DDS (consumption of 4–5 food groups) and high DDS (consumption of 6 and above food groups)^17^. Mothers who had reported exclusive breastfeeding below 6 months were considered as events and those who exclusively breastfeed to 6 months and beyond were taken as censored.

### Data collection procedure

Pretested and structured questionnaires were used for data collection. The questionnaire was partly adapted from the Ethiopian demographic survey (EDHS)^17^ and a review of similar literature^13,14,18–20^. Data were collected by four diploma nurses and supervised by three BSc nurses. A two days comprehensive training was given to data collectors and supervisors. The questionnaire was first prepared in English and then translated into Amharic (the local language), and back into English to ensure consistency. Data collection was on working days for unemployed mothers (Monday to Friday). Employed mothers were interviewed during weekends and before they go to their workplaces or at their workplaces for some employed mothers.

### Data processing and analysis

The data were doubly entered to Epi data 3.1 and exported to SPSS version 23 for analysis. The data were cleaned by checking outliers and missing values. Percentages were used to described categorical variables while mean and standard deviation (± SD) or medians and interquartile ranges (IQR) were used to described continuous variables.

Survival analysis using Kaplan–Meier was done to assess the difference in duration of exclusive breastfeeding among employed and unemployed mothers. The log-rank test was used to assess the presence of a significant difference in survival status of exclusive breastfeeding between employed and unemployed mothers. Bivariate Cox proportional hazards model was used to identify determinants of time to stop exclusive breastfeeding. The Association of each covariate with the duration of exclusive breastfeeding was assessed using the Bivariate Cox proportional hazards model after the proportional hazard assumption was checked using a log–log(st)plot. Those variables with P < 0.25 in the bivariate Cox regression model were entered in the multivariate Cox proportional hazards model to measure the effect of each variable on the hazard function after adjusting for the effects of other variables included in the model. Multivariate Cox proportional hazards model was fitted with forwarding likelihood ratio (forward LR). Variables with P < 0.05 in multiple Cox regression analysis were identified as determinants of cessation of exclusive breastfeeding before 6 months. Adjusted Hazard Ratio (AHR) with 95% Confidence Interval (CI) and p < 0.05 were used to declare the level of significance.

### Ethics approval and consent to participate

Ethical approval was obtained from the institutional review board of Jimma University. Oral informed consent was taken with full information including the objectives of the study, selection criteria, confidentiality and benefits of the study. Informed consent obtained from a parent and/or caregiver’s of the infants. All methods were carried out in accordance with relevant guidelines and regulations.

## Results

### Socio-demographic characteristics of the respondents

Four hundred twenty-six (426) respondents were successfully interviewed with a response rate of 100%. The mean (± SD) age of mothers was 30.1 (0.9) years and the mean (SD) age of infants was 8.4 (0.8) months. Among employed mothers, (61%) were government employed, (29.6%) were in the highest wealth quantile and (17.4%) were in the lowest wealth quantile. Conversely (9.9%) of unemployed mothers were in the highest wealth quantile and (17.4%) were in the lowest quantile of wealth (Table 1).

**Table 1:** Socio-demographic characteristics of the study subjects according to the employment status of mothers of 6–12 months old infants at Debre tabor town, Northwest Ethiopia, 2019.

Variables	Employment status		
	Employed (n = 213), n (%)	Unemployed (n = 213), n (%)	Total (n = 426)
Sex of child			
Female	115 (54)	97 (45.5)	212 (49.8)
Male	98 (46)	116 (54.5)	214 (50.2)
Age of child			
6–8 months	90 (42.3)	96 (45.1)	186 (43.7)
9–10 months	68 (31.9)	70 (32.9)	138 (32.4)
11–12 months	55 (25.8)	47 (22.1)	102 (23.9)
Birth order			
First	98 (46)	84 (39.4)	182 (42.7)
Second	62 (29.1)	60 (28.2)	122 (28.6)
Third	36 (16.9)	42 (19.7)	78 (18.3)
Fourth and above	17 (8)	27 (12.7)	44 (10.3)
Maternal age			
15–20	7 (3.3)	7 (3.3)	14 (3.3)
21–25	33 (15.5)	55 (25.8)	88 (20.7)
26–30	108 (50.7)	86 (40.4)	194 (45.5)
31–35	47 (22.1)	39 (18.3)	86 (20.2)
Greater than 35	18 (8.5)	26 (12.2)	44 (10.3)
Parity			
Primipara	79 (37.1)	84 (39.4)	163 (38.3)
Multipara	134 (62.9)	129 (60.6)	263 (61.7)
Marital status			
Married	187 (87.8)	202 (94.8)	389 (91.3)
Single	5 (2.3)	1 (0.5)	6 (1.4)
Divorced	16 (7.5)	9 (4.2)	25 (5.9)
Widowed	5 (2.3)	1 (0.5)	6 (1.4)
Occupation			
Housewife		213 (100)	213 (50)
Merchant	58 (27.2)		58 (13.6)
Government employee	130 (61.0)		130 (30.5)
Daily labourers	17 (8.0)		17 (4)
Others	8 (3.8)		8 (1.9)
Wealth index			
Lowest quantile	37 (17.4)	37 (17.4)	74 (17.4)
Second quantile	44 (20.7)	75 (35.2)	119 (27.9)
Middle quantile	25 (11.7)	38 (17.8)	63 (14.8)
Fourth quantile	44 (20.7)	42 (19.7)	86 (20.2)
Highest quantile	63 (29.6)	21 (9.9)	84 (19.7)

### Health service and obstetrics related factors

The majority of respondents (75.8%) reported that they had four times and above ANC visits. During this visit (95.5%) of total mothers got information about exclusive breastfeeding. However, (42.5%) of mothers who attended PNC were informed about EBF during PNC.

Among employed mothers (78.4%) had four times and above ANC visits and (25.8%) of them were informed about EBF during PNC. Regarding unemployed mothers, (73.2%) of them had four times and above ANC visits and (59.2%) were informed about EBF during PNC. The majority of the respondents, (99.5%), delivered their child to a health facility. From the total mothers, 95.5% of them had birth attendant of health professional (Table 2).

**Table 2:** Health service and obstetric related factors of employed and unemployed mothers at Debre tabor town, Northwest Ethiopia, 2019.

Variables	Employment status		
	Employed (n = 213) n (%)	Unemployed (n = 213) n (%)	Total
Time of ANC follow up			
Once	5 (2.3	1 (0.5)	6 (1.4)
Twice	11 (5.2)	12 (5.6)	23 (5.4)
Three times	30 (14.1)	44 (20.7)	74 (17.4)
Four times and above	167 (78.4)	156 (73.2)	323 (75.8)
Get health information about EBF at ANC			
Yes	201 (94.5)	206 (96.7)	407 (95.5)
No	12 (5.6)	7 (3.3)	19 (4.5)
PNC services			
No	158 (74.2)	87 (40.8)	245 (57.5)
Yes	55 (25.8)	126 (59.2)	181 (42.5)
Get health information about EBF at PNC			
No	41 (74.2)	51 (40.8)	104 (57.5)
Yes	14 (25.8)	75 (59.2)	77 (42.5)
Place of delivery			
At home	–	2 (0.9)	2 (0.5)
At health facility	213 (100)	211 (99.1)	424 (99.5)
Birth attendant			
Health professional	213 (100)	211 (99.1)	424 (99.5)
Non-health professional	–	2 (0.9)	2 (0.2)
Mode of delivery			
Vaginal	200 (93.9)	207 (97.2)	407 (95.5)
Cesarean section	13 (6.1)	6 (2.8)	19 (4.5)
EBF information during delivery			
No	157 (73.7)	87 (40.8)	244 (57.3)
Yes	56 (26.3)	126 (59.2)	182 (42.7)

### Dietary diversity of mothers

Among employed mothers, (58.2%) have dietary diversity of low/consumption of less than four food groups. However, only (1.9%) have dietary diversity of high/consumption of 6 or more food groups. On the other hand, (74.2%) of unemployed mothers had dietary diversity of low and (25.8%) of them had DDS of medium (consumption of 4–5 food groups). None of the unemployed mothers had dietary diversity of high (Table 3**).**

**Table 3:** Dietary diversity of employed and unemployed mother at Debre-tabor town, Northwest Ethiopia, 2019.

	Employment status		
	Employed (n = 213) n%	Unemployed (n = 213) n %	Total
Dietary diversity of mothers			
Low/less than four food groups/	124 (58.2)	158 (74.2)	282 (66.2)
Medium/4–5 food groups/	85 (39.9)	55 (25.8)	140 (32.9)
High/6 and more food groups	4 (1.9)		4 (0.9)

### Breastfeeding practice

Four hundred ten (96.2%) of the respondents give breast milk to their children within the first hour. The frequency of breastfeeding was nine times and greater per day among 239 (56.1%) of the respondents. Among employed mothers 192 (90.1%) of the employed mothers gave Colostrum to their child, 57 (26.8%) of the breastfed their child greater than or equal to 9 times per 24 h and 87 (40.8%) of them got advice about exclusive breastfeeding by their husbands. On the other hand, among unemployed mothers, 195 (91.5%) of them gave Colostrum to their child, 130 (61%) of them breastfed their child greater than or equal to 9 times per 24 h and 61 (28.6%) of them got advice about exclusive breastfeeding by their husbands. From a total of employed mothers 57 (26.8%) of them got family support to exclusively breastfed their child, while 165 (77.5%) of unemployed mothers got family support to exclusively breastfed their child (Table 4).

**Table 4:** Breastfeeding practice and perception of mothers at Debre tabor town, Northwest Ethiopia, 2019.

Variables	Employment status		Total
	Employed (n = 213) n%	Unemployed (n = 213) n %	
Initiation of breastfeeding			
Within 1 h	203 (73.2)	207 (97.2)	410 (96.2)
Greater than 1 h	10 (26.8)	6 (2.8)	16 (3.8)
Colostrum feeding			
No	21 (9.9)	18 (8.5)	39 (8.5)
Yes	192 (90.1)	195 (91.5)	390 (91.5)
Reason of colostrum avoidance			
Lack of knowledge	15 (7.0)	12 (5.6)	27 (6.3)
Others	6 (2.8)	6 (2.8)	12 (2.8)
Frequency of breastfeeding per day			
Less than 9 times	156 (73.2)	83 (39)	239 (56.1)
Greater than or equal to 9 times	57 (26.8)	130 (61)	187 (43.9)
Time to give breast milk			
On maternal demand	70 (32.9)	91 (42.7)	161 (37.8)
When the bay cries	13 (6.1)	26 (12.2)	39 (9.2)
On schedule	38 (17.8)	8 (3.8)	46 (10.8)
Maternal demand and when baby cry	91 (42.7)	88 (41.3)	179 (42)
Others	1 (0.5)	–	1 (0.2)
Family support to exclusively breastfed			
No	156 (73.2)	48 (22.5)	204 (47.9)
Yes	57 (26.8)	165 (77.5)	222 (52.1)
Husband role to EBF			
Advice on EBF	87 (40.8)	61 (28.6)	149 (35)
Economic support	31 (14.6)	74 (34.7)	114 (26.8)
Has no role	80 (37.6)	63 (29.6)	133 (31.2)
Do not know	15 (7)	15 (7)	30 (7)
Religious leaders encouragement to EBF			
No	206 (96.7)	201 (94.4)	407 (95.5)
Yes	7 (3.3)	12 (5.6)	19 (4.5)
Best food for infants in the first six months			
Breast milk only	211 (99.1)	213 (100)	424 (99.5)
Others	2 (0.9)		2 (0.5)
Breast milk prevents disease			
No		4 (1.9)	4 (0.9)
Yes	213 (100)	209 (98.1)	422 (99.1)

### The median time to stop exclusive breastfeeding among employed and unemployed mothers

**Figure 1:**
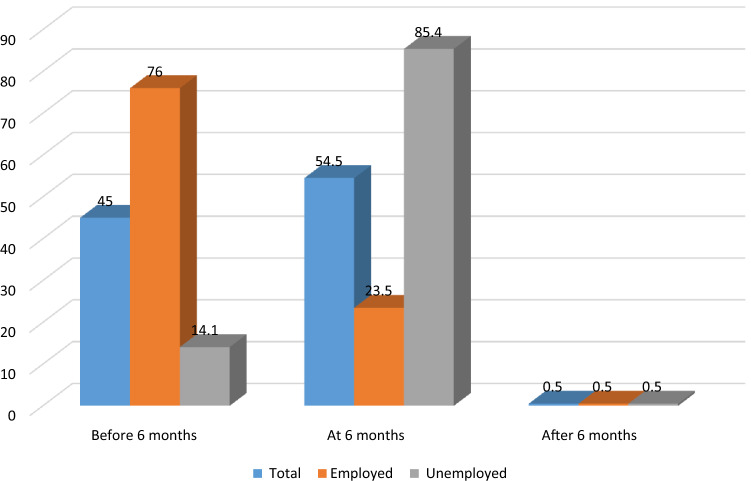
Percentage of cessation of EBF by the duration of time among employed and unemployed mothers in Debre tabor town, South Gondar Zone, North West Ethiopia, 2019.

From the total of women, 45% of them cease exclusive breastfeeding before 6 months. Among employed mothers, 76% of them cease exclusive breastfeeding before 6 months, while 23.5% of them cease exclusive breastfeeding at 6 months. Conversely, 14.1% of unemployed mothers stop exclusive breastfeeding before 6 months, while 85.4% of them stop exclusive breastfeeding at 6 months (Fig. 1).

**Figure 2:**
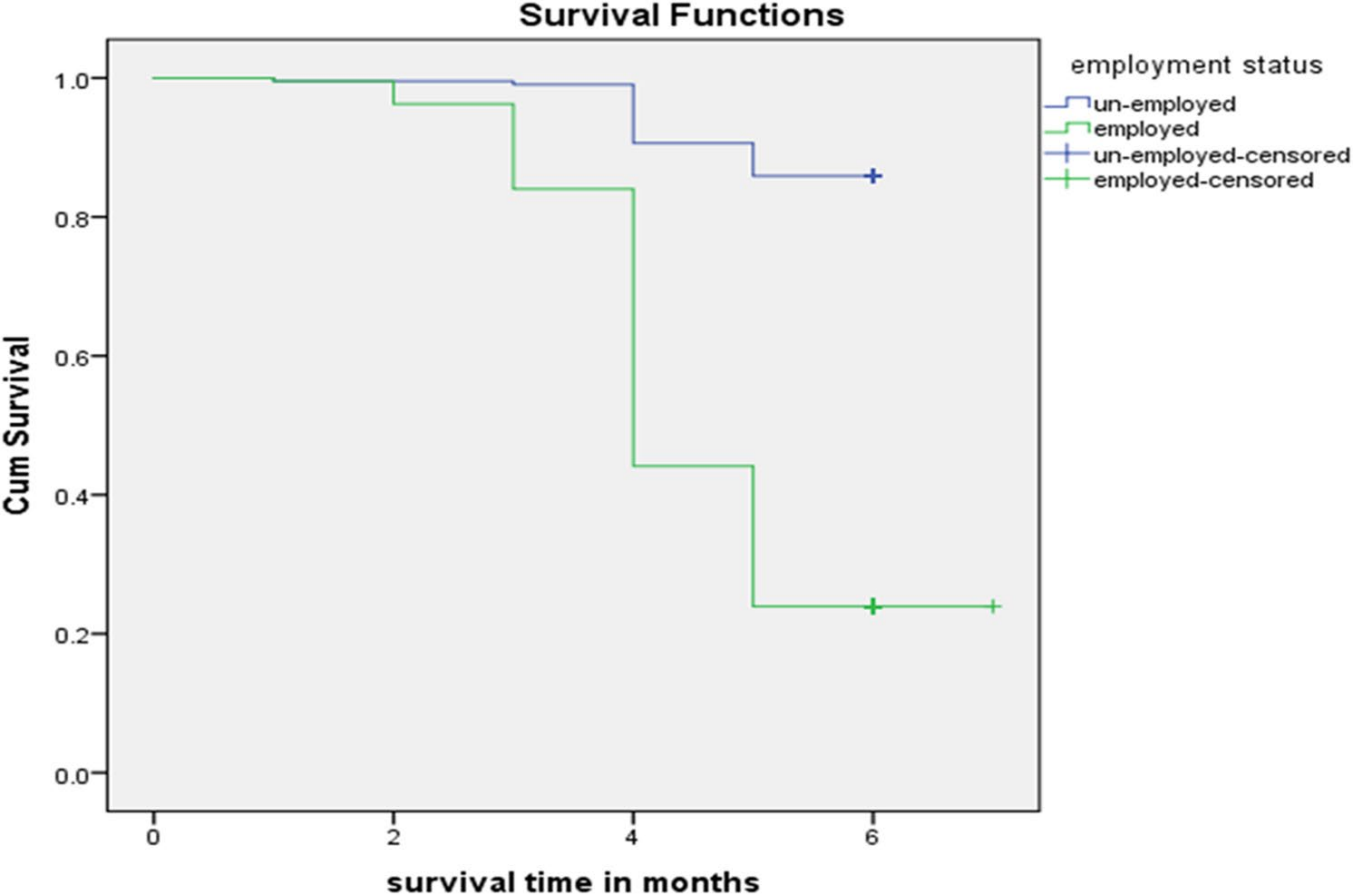
Kaplan–Meier cumulative survival probability functions of EBF for children of employed and unemployed mothers in Debre tabor town, South Gondar Zone, North West Ethiopia, 2019.

The median duration of exclusive breastfeeding for employed mothers was 4 months, while 6 months for unemployed mothers. The minimum and maximum duration of exclusive breastfeeding were 1 and 7 months respectively for both children of employed and unemployed mothers. The median duration of exclusive breastfeeding for all children was 6 months. The cumulative proportion of survival probability up to 3 months on exclusive breastfeeding was 96% for employed mothers and 100% for unemployed mothers. While it was 98% for total mothers by considering cessation of exclusive breastfeeding before 6 months as an event. The cumulative proportion of survival probability up to 4 months on EBF was 84% for employed mothers and 99% for unemployed mothers. This is the time when the cumulative survival probability started to be significantly different among employed and unemployed mothers. The cumulative survival probability of up to 5 months on exclusive breastfeeding was 44% for employed mothers and 91% for unemployed mothers. While it was 67% for total mothers. The cumulative proportion of survival probability up to 6 months on exclusive breastfeeding for total children was 55% as illustrated by the life table (Table 5). The cumulative proportion of survival probability up to 6 months on exclusive breastfeeding was higher for children of unemployed mothers compared to children of employed mothers accounting for 86% and 24% respectively. The difference was statistically significant on Log-rank (Cox-Mantel) test < 0.001 (Fig. 2**).**

**Table 5:** Life table for exclusive breastfeeding duration for 6–12 months’ age children of employed and unemployed mothers at Debre tabor Town, Northwest Ethiopia, 2019.

	EBF interval (months)	Interval start time	Number entering interval	Events	Cumulative events	Remaining cases	Proportion of surviving	Surviving at the end of the interval	Hazard rate
Un-Employed	0–1	0	213	0	0	213	1.00	1.00	0.00
	1–2	1	213	1	1	212	1.00	1.00	0.00
	2–3	2	212	0	1	212	1.00	1.00	0.00
	3–4	3	212	1	2	211	1.00	0.99	0.00
	4–5	4	211	18	20	193	0.91	0.91	0.09
	5–6	5	193	10	30	183	0.95	0.86	0.05
	6–7	6	183	0	30	183	1.00	0.86	0.00
Employed	0–1	0	213	0	0	213	1.00	1.00	0.00
	1–2	1	213	1	1	212	1.00	1.00	0.00
	2–3	2	212	7	8	205	0.97	0.96	0.03
	3–4	3	205	26	34	179	0.87	0.84	0.14
	4–5	4	179	85	119	94	0.53	0.44	0.62
	5–6	5	94	43	162	51	0.54	0.24	0.59
	6–7	6	51	0	162	51	1.00	0.24	0.00
Total	0–1	0	426	0	0	426	1.00	1.00	0.00
	1–2	1	426	2	2	424	1.00	1.00	0.02
	2–3	2	424	7	9	417	0.98	0.98	0.07
	3–4	3	417	27	36	390	0.94	0.92	0.30
	4–5	4	390	103	139	287	0.74	0.67	0.20
	5–6	5	287	53	192	234	0.82	0.55	0.00
	6–7	6	234	0	162	234	1.00	0.55	0.00

### Determinants of exclusive breastfeeding

In bivariate cox proportional hazards model for total mother’s candidates for multivariate cox regression model were the computed (Table 6). On the multivariate cox proportional hazards model, employment status, parity of mothers, family support to exclusive breastfeeding and EBF information at PNC were statically significant determinants of cessation of exclusive breastfeeding before 6 months. The hazard of cessation of EBF among employed mothers was 3.7 times that of unemployed mothers (AHR = 3.77, 95% CI 2.39–5.9, P < 0.001). Primipara mothers were 1.5 times more likely to cease EBF before 6 months as compared to multipara mothers (AHR = 1.5, 95% CI 1.14–2.04, P = 0.005). Mothers who had no family support to exclusively breastfed were 3.9 times more likely to cease EBF before 6 months as compared to mothers who had family support to EBF (AHR = 3.99, 95% CI 1.9–8.3, P < 0.001). The hazard of cessation of EBF before 6 months among mothers who did not get EBF counselling at PNC was 7.7 times as compared to mothers who got EBF counselling at PNC (AHR = 7.76, 95% CI 2.99–20.1, P < 0.001) (Table 7).

**Table 6:** Bivariate cox proportional hazards model of cessation of EBF among women who have 6–12 months’ age child in Debretabor town, North West Ethiopia, 2019.

Variables	CHR	95% CI	P value
Employment status			
Unemployed	1		
Employed	7.41	5.01–10.968	< 0.001
Birth order			
First	0.499	0.353–0.705	< 0.001
Second	0.357	0.224–0.569	< 0.001
Third	0.347	0.192–0.630	0.001
Fourth and above	1		
Maternal educational status			
No education	1		
Primary	0.215	0.126–0.368	< 0.001
Secondary	0.316	0.207–0.483	< 0.001
College and above	0.363	0.231–0.571	< 0.001
Husband educational status			
No education	1		
Primary	0.265	0.109–0.646	0.004
Secondary	0.308	0.174–0.544	< 0.001
College and above	0.406	0.25–0.656	< 0.001
Occupation			
Housewife	1		
Merchant	5.010	3.055–8.216	< 0.001
Government employee	9.608	6.403–14.420	< 0.001
Daily laborer	6.629	3.39–12.964	< 0.001
Others	.896	0.122–6.567	0.94
Frequency of breast feeding			
Greater than or equal to 9 times	1		
< 9 times	16.1	8.96–28.93	< 0.001
Family support to EBF			
Yes			
No	25.8	14.34–46.47	< 0.001
EBF counseling during PNC			
Yes	1		
No	32.3	14.3–73	< 0.001
EBF counseling during delivery			
Yes	1		
No	23.9	11.8–48.77	< 0.001
Best food for < 6 months children			
Breast milk only	1		
Others	3.247	0.8–13	0.098
Recommended duration of EBF			
< 6 months	1		
6 months	0.344	0.24–0.49	< 0.001
> 6 months	0.122	0.03–0.5	0.004
Role of husband to EBF			
Advice on EBF			
Give economic support	0.596	0.38-.92	0.020
Has no role	1.910	1.37–2.64	< 0.001
Do not know	1.041	0.57–1.89	0.896
Breast milk sufficiency in the first 6 months			
Yes			
No	1.416	1.006–1.994	0.046
Religious fathers encouragement to EBF			
Yes			
No	2.52	0.93–6.80	0.067
Wealth index			
Lowest quantile	0.82	0.54–1.26	0.373
Second quantile	0.60	0.40–0.89	0.013
Middle quantile	0.49	0.30–0.828	0.007
Fourth quantile	0.71	0.47–1.09	0.120
Highest quantile	1		
Parity			
Primipara	1.77	1.33–2.345	< 0.001
Multipara	1

**Table 7:** Bivariate and multivariable cox proportional hazards model predicting the hazards of cessation of EBF among women who have 6–12 months’ age child in Debre tabor town, South Gondar zone, Northwest Ethiopia, 2019.

Variables	CHR	95%CI	P-value	AHR	95%CI	P-value
Employment status						
Employed	7.4	5.01–10.96	< 0.001	3.77	2.39–5.9	< 0.001
Unemployed	1					
Family support						
Yes	1					
No	25.8	14.34–46.47	< 0.001	3.99	1.9–8.3	< 0.001
Get EBF information at PNC						
Yes	1					
No	32.3	14.3–73.0	< 0.001	7.76	2.99–20.1	< 0.001
Perception on the recommended duration of EBF						
< 6 months	1					
6 months	0.344	0.24–0.494	< 0.001	0.55	0.37–0.80	0.002
> 6 months	0.12	0.029–0.51	0.004	0.20	0.05–0.85	0.029
Parity						
Primipara	1.77	1.33–2.35		1.52	1.14–2.04	0.005
Multipara	1					

## Discussion

The findings of the study revealed that employed mothers were 3.7 times more likely to cease exclusive breastfeeding before 6 months. Duration of paid maternity leave was an associated factor for cessation of exclusive breastfeeding before 6 months among employed mothers. Family support and perceived breast milk adequacy were associated factors with cessation of exclusive breastfeeding before 6 months among unemployed mothers. For total mothers, employment status, parity, family support of breastfeeding, counselling of exclusive breastfeeding during postnatal care and maternal perception of exclusive breastfeeding duration were the independent significant determinant factors of cessation of EBF before 6 months.

In this study employed mothers were more likely to not exclusively breastfeed for 6 months compared to unemployed women. This difference was comparable with other studies conducted in Ethiopia^13,14^, Saudi Arabia^21^, and Canada^22^ in which the exclusive breastfeeding rate was higher among the unemployed women compared to employed women. Furthermore, employed mothers may likely return to work early after giving birth if necessary, support to continue exclusive breastfeeding is not provided by employers. Hence separation from their babies, and additional difficulties in expressing and storing milk results in an inability to maintain exclusive breastfeeding among employed women^23^. On the other hand, unemployed mothers get a longer time to stay with their children^24^, hence early termination of exclusive breastfeeding was observed more in employed mothers than in unemployed mothers.

Furthermore, employment rules and regulations, such as maternity leave less than 6 months (4 months in the Ethiopian context), compromising exclusive breastfeeding. This problem is exacerbated by a lack of child care facilities and other supportive work environments. Thus, women who return to work often face difficulties in continuing breastfeeding^25,26^.

According to this study, among employed mothers, the duration of paid maternity leave was one of the determinants of cessation of exclusive breastfeeding before 6 months. The hazard of cessation of exclusive breastfeeding before 6 months for employed mothers who had paid maternity leave of 4 months only was 7 times higher than employed (working) mothers who had paid maternity leave of greater than 4 months. This result was supported by evidence from 38 low- and middle-income countries, in which a one-month increase in the legislated duration of paid maternity leave was associated with a 5.9% increase in exclusive breastfeeding and a 2.2-month increase in breastfeeding duration^13^.

The median duration of exclusive breastfeeding in this study was 6 months for all women, which was comparable with the studies conducted in Ethiopia, Gurage zone^27^, Awi zone^28^ and in India^29^ in which the median duration of exclusive breastfeeding were 6 months, 6.06 months and 6 months respectively. However, this finding is higher than the EDHS 2016 report, in which the median duration of exclusive breastfeeding was 3.1 months. The possible reason for this could be due to national representative data of EDHS and the fact that this study was conducted in an urban setting. Moreover, This finding is also greater than the median duration of exclusive breastfeeding in Kongo, Sri Lanka, China and Brazil^30–33^. This difference could be a result of the difference in the study settings.

The cumulative proportion of survival probability of exclusive breastfeeding for six for total children was 55%. This result was consistent with a study conducted in Ethiopia, Awi zone which reported a cumulative survival probability for exclusive breastfeeding for 6 months of 53%^20^. However, this finding is lower than another study conducted in Ethiopia that reported a 78.1% probability of exclusive breastfeeding for 6 months^19^. This difference could be due to the fact that the study participants for the study were from urban and rural settings compared to this study which was done in urban women. Hence, due to the inclusion of rural mothers who usually exclusively breastfeed longer the survival probability of up to 6 months will be greater. Furthermore, since more employed mothers are found in an urban area than rural and stay a long time away from their home for jobs.

Being primipara, lack of family support, not receiving information about exclusive breastfeeding during postnatal care visit and maternal perception that exclusive breastfeeding duration should be less than 6 months were other independent determinant factors that significantly increase the risk of terminating exclusive breastfeeding before 6 months. In this study, primipara mothers were 1.5 times more likely to stop exclusive breastfeeding than multipara mothers. This result is supported by a study conducted in Gondar, in which mothers who have three or more children were 3.5 times more likely to breastfeed exclusively than those who had one or two children. This result is also comparable with a study conducted in the USA, Hershey, in which multiparous mothers had a significantly lower hazard of stopping exclusive breastfeeding as compared to multiparous mothers^33^. This could be due to the fact that though breastfeeding is a natural act, it is also a learned behaviour. As a result, multipara mothers are more experienced and knowledgeable on the advantage of exclusive breastfeeding^13,34^.

Family support in the form of encouragement was also a significant determinant of the duration of exclusive breastfeeding. Mothers who had family support for exclusive breastfeeding were less likely to stop exclusive breastfeeding before 6 months. The hazard of mothers who had no family support for exclusive breastfeeding was 3.48 times as compared to mothers who had family support. This result was comparable with a study conducted in Indonesia, in which mothers who had good family support were 2.8 times more likely to practice exclusive breastfeeding^35^. Another study in Indonesia supported this result, in which respondents were dominated by their mother's decisions and there is a significant relationship found between the level of partner support and practice of exclusive breastfeeding^36^.

Postnatal counselling on exclusive breastfeeding was also significantly associated with the cessation of exclusive breastfeeding in this study. The hazard of mothers who were not informed about exclusive breastfeeding in their post-natal care visits was 3.9 times more likely to cease exclusive breastfeeding before 6 months as compared to mothers who were informed about exclusive breastfeeding during postnatal care visit. This result was higher than the study conducted in Ethiopia, Awi zone, in which the hazard of cessation of exclusive breastfeeding among mothers who were informed about EBF during postnatal was 1.94 times as compared to mothers who were not informed^20^. This deference might be due to deference in counselling services of deferent areas. This result was lower than the study conducted in Ethiopia, Gurage zone, in which the hazard of cessation of exclusive breastfeeding before 6 months among mothers who were not informed /counselled during postnatal was 5.1 times as compared to mothers who were counselled during their postnatal visits^19^. The possible reason for this difference could be due to study design and study participants of both urban and rural.

The hazard of cessation of exclusive breastfeeding among mothers who thought the recommended duration of exclusive breastfeeding < 6 months was higher than mothers who thought the recommended duration of exclusive breastfeeding 6 months. This is in agreement with a study conducted in Saudi Arabia, in which lack of awareness of exclusive breastfeeding was one of the factors limiting breastfeeding^37^.

## Limitation of the study

Recall bias might affect the response since the way of the interview were by back history and experience. To decrease the recall bias, data were collected by interviewing the mothers to give their response about different types of events. Using full month recording for the duration of exclusive breastfeeding than using specific date helps respondents to easily remember the time and reduces recall bias.

## Conclusion

In this study, a significant proportion of women cease exclusive breastfeeding before 6 months. The median duration of exclusive breastfeeding was shorter among employed mothers than un-employed mothers. The difference was statistically significant. It is clear from this study that cessation of exclusive breastfeeding was associated with the duration of paid maternity leave for employed mothers. Family support and perceived adequacy of breast milk was associated with cessation of exclusive breastfeeding before 6 months among unemployed mothers. Being primipara, lack of family support, absence of post-natal counselling, and maternal perception on the recommended duration of exclusive breastfeeding were the significant determinant of the cessation of exclusive breastfeeding before 6 months among both groups of mothers.

## Data Availability

The data used and/or analyzed during the current study are available from the corresponding author on reasonable request.

## Abbreviations

EBF: Exclusive breastfeeding
IYCF: Infant and young child feeding
